# Stearoyl-ACP Δ^9^ Desaturase 6 and 8 (GhA-SAD6 and GhD-SAD8) Are Responsible for Biosynthesis of Palmitoleic Acid Specifically in Developing Endosperm of Upland Cotton Seeds

**DOI:** 10.3389/fpls.2019.00703

**Published:** 2019-05-31

**Authors:** Baoling Liu, Yan Sun, Jinai Xue, Xue Mao, Xiaoyun Jia, Runzhi Li

**Affiliations:** Institute of Molecular Agriculture and Bioenergy, Shanxi Agricultural University, Jinzhong, China

**Keywords:** cotton, ACP-Δ^9^ desaturase, palmitoleic acid, endosperm, substrate specificity

## Abstract

Palmitoleic acid (16:1Δ^9^) is one kind of ω-7 fatty acids (ω-7 FAs) widely used in food, nutraceutical and industry. However, such high-valued ω-7 FA only has a trace level in mature seeds of cotton and other common oil crops. We found that palmitoleic acid (>10.58 Mol%) was specially enriched in developing cotton endosperm which is disappeared in its mature seed. The present study was conducted to investigate the mechanism underlying high accumulation of palmitoleic acid in developing endosperm but not in embryo of upland cotton (*Gossypium hirsutum* L.) seed. Of 17 stearoyl-ACP Δ^9^ desaturases (SAD) gene family members identified in upland cotton, six GhSADs may specifically work in the desaturation of palmitic acid (16:0-ACP) to produce palmitoleic acid (16:1Δ^9^-ACP), which were revealed by examining the key amino acids in the catalytic center and their *cis*-elements. Gene expression analysis showed that spatial patterns of these *GhSADs* were different in developing ovules, with *GhA-SAD6* and *GhD-SAD8* preferentially expressed in developing endosperms. Functional analysis by transient expression in *Nicotiana benthamiana* leaves and genetic complementary assay using yeast mutant *BY4389* strain unable to synthesize unsaturated fatty acids demonstrated that GhA-SAD6 and GhD-SAD8 have strong substrate specificity for 16:0-ACP. In contrast, GhA-SAD5 and GhA-SAD7 exhibited high specific activity on 18:0-ACP. Taken together, these data evidence that GhA-SAD6 and GhD-SAD8 are responsible for making palmitoleic acid in developing cotton endosperms, and provide endogenous gene targets for genetic modification to enrich ω-7 FAs in cotton seed oil required for sustainable production of functionality-valued products.

## Introduction

Cotton (*Gossypium* sp.) is widely cultivated in the world as an important industrial crop, providing excellent fiber and protein plus oil. Particularly, cottonseed oil is an important vegetable oil and broadly used in food/catering, clean biofuel and other industries (Jiao et al., 2013; Sturtevant et al., 2017). Fatty acid composition and its content determine the value and utilization of vegetable oil. The conventional cottonseed oil mainly consists of 26% palmitic acid (16:0), 58% linoleic acid (18:2^Δ9,12^), 13% oleic acid (18:1Δ^9^) and 2% stearic acid (18:0) (Zhao et al., 2018). Since linoleic acid is oxidatively unstable, cottonseed oil is easily oxidized and rancid, and thus it normally needs partial hydrogenation before food applications. However, byproducts of this process are *trans-*fatty acids, which are harmful for human health (Ginter and Simko, 2016). Saturated palmitic acid is not recommended for direct edible use because of its detrimental effects on human health despite of its highly oxidative stability (Shang et al., 2017). Monounsaturated fatty acids (MUFAs) such as oleic acid and palmitoleic acid (16:1Δ^9^) are health-promoting fatty acids, but they are in tiny content in cottonseed oil. Therefore, the development of designer cottonseed oil with nutritionally desirable fatty acids has been an attractive object to meet oil marketing demand.

Omega-7 fatty acids (ω-7 FAs), are monounsaturated lipids with a double-bond seven atoms away from the methyl end of the acyl chain, including palmitoleic acid (16:1Δ^9^), and its elongation products, vaccenic acid (*cis*-18:1Δ^11^) and paullinic acid (*cis*-20:1Δ^13^). ω-7 FAs are high-valued in human healthcare, but also in multiple industrial applications, ranging from the precursor of 1-octene for polyethylene to the feedstock of biodegradable lubricant and biodiesel (Curb et al., 2000; Misra et al., 2010; Mozaffarian et al., 2010; Nguyen et al., 2010, 2015). Unfortunately, such valued ω-7 FAs are very low abundance in commercialized field oilseeds like cotton and soybean (*Glycine max*). Only a few of uncommercialized plants can synthesize and accumulate much high level of ω-7 FAs in their seeds (Wu et al., 2012). For example, contents of ω-7 FAs are high up to 64%, 30%, and 32% in the mature seeds of cat’s claw (*Doxantha unguis-cati* L.), macadamia (*Macadamia integrifolia*), and sea buckthorn (*Hippophae rhamnoides*), respectively (Cahoon et al., 1998; Gummeson et al., 2000; Kallio et al., 2002). Interestingly, ω-7 FAs were found to be highly concentrated in the endosperm (aleurone) but not in the embryo in the seeds of *Arabidopsis thaliana* and rapeseed (*Brassica napus*), with 15 mol% in Arabidopsis and 35% in rapeseed, respectively (Bryant et al., 2016). It is noteworthy that the endosperm is reduced to a single cell layer of residues surrounding the embryo in these mature seeds (Penfield et al., 2004; Li et al., 2006). Such a thin peripheral aleurone and seed coat are tightly stuck together, and consequently are stripped off the embryo during the dehulling process before oil extraction. The detailed molecular mechanism responsible for such high biosynthesis of ω-7 FAs in the resource plants but not in the embryo of common oilseeds remains to be elucidated despite a number of enzymes and transcriptional factors were found to play important roles in formation of ω-7 FAs (Bryant et al., 2016; Troncoso-Ponce et al., 2016).

In plants, fatty acyl-ACPs of different chain lengths and saturation levels are initially biosynthesized in plastid. Then, fatty acyl-ACPs are catalyzed by fatty acyl-ACP thioesterases to release free fatty acids chains, which are transported from plastid via cytosol into endoplasmic reticulum (ER) for functional modifications, such as acyl chain elongation and further desaturation. Finally, fatty acids are channeled into triacylglycerol (TAG) by cascade acylation reactions. Alternatively, fatty acids could also be integrated into phosphatidylcholine (PC) and other acyl carrier molecules.

It is believed that ω-7 FAs are produced by Δ^9^ desaturation of palmitic acid (16:0) to form palmitoleic acid (16:1Δ^9^), which can then be elongated (Cahoon et al., 1998). Such Δ^9^ desaturation is catalyzed by acyl-ACP Δ^9^ desaturase (AAD, EC 1.14.99.6) family (Parvini et al., 2016). The archetype for this family is the stearoyl-ACP Δ^9^ desaturase (named shortly as SAD) which normally desaturates stearic acid (18:0) to form oleic acid (18:1Δ^9^, *cis*-ω9) at high efficiency in plants. However, various AAD isoforms also exhibit different levels of substrate specificity on 16:0-ACP (McKeon and Stumpf, 1982). Those AAD isoforms with preference on 16:0-ACP instead of 18:0-ACP as substrate are also named as palmitoyl-ACP Δ^9^ desaturases (PADs), and some of which were isolated from several high accumulators of ω-7 FAs (Cahoon et al., 1997, 1998).

It is therefore recognized that the specific activity of SAD enzymes for substrates determines the ratio of 16:1Δ^9^ to 18:1Δ^9^ and their derivatives (ω-7 and ω-9 FAs) in plant seeds. The key amino acids located in the catalytic domain were reported to play important roles for the substrate specificity of SAD enzymes (Whittle and Shanklin, 2001; Zhang Y. et al., 2015), but knowledge is still limited for the molecular determination of such substrate specificity. Recently, Arabidopsis AtAAD2 and AtAAD3 were identified as the major contributors for the synthesis of ω-7 FAs in the aleurone, though seven AtAAD family members were detected in Arabidopsis genome (Bryant et al., 2016; Troncoso-Ponce et al., 2016).

As described above, cottonseeds contain high level of 16:0 (∼25%) and 18:2Δ^9,12^ (>50%), but little 16:1Δ^9^ (<1%) and 18:1Δ^11^ (<1%), showing that SAD enzymes responsible for Δ^9^ desaturation in upland cotton may have very higher selectivity for 18:0-ACP than for 16:0-ACP. However, RNAi down-regulation of *KASII* resulted in remarkable increase of 16:1Δ^9^ and 18:1Δ^11^ in cotton seeds by 10% and 1.5%, respectively, indicating the existence of SAD isoforms having high specific activity to catalyze 16:0-ACP to 16:1-ACP (Liu et al., 2017). More importantly, we examined fatty acid profiles in separate tissues from cotton seeds, revealing that a certain amount of 16:1Δ^9^ were concentrated in developing endosperm but not in embryo. Overall, there should be existed some SAD members responsible for biosynthesis of 16:1Δ^9^ in upland cotton. How many members of SAD family are in allotetraploid upland cotton? Is there any SAD isoform having a higher substrate specificity toward 16:0-ACP or 18:0-ACP? If so, what are the distinct structure features between 16:0- and 18:0-specific SAD isoforms? How they work coordinately in controlling the biosynthesis of ω-7 and ω-9 FAs in cotton seeds? Particularly, which members of SAD family in cotton are responsible for ω-7 FA enrichment in developing endosperm but not in embryo? Since endosperm would be vanished from the mature seeds, what physiological roles are ascribed to ω-7 FAs exclusively accumulated in developing endosperm? All these questions need to be addressed.

Here, a genome-wide characterization was performed to identify candidate SAD family members in upland cotton (*Gossypium hirsutum* L.), a naturally occurring allotetraploid (2n = 4x = (AD)_2_ = 52) produced by interspecific hybridization of A- and D-genome diploid progenitor species (Zhang T. et al., 2015). Analysis on the key amino acids in the functional domain of GhSAD members and their *cis*-elements as well as 3D structure were conducted to identify the putative 16:0-specific GhSADs. And then, the temporal-spatial expression patterns of these *GhSAD* genes were examined by both RNA-seq data and qRT-PCR in various cotton tissues and developing seeds. Subsequently, for functional analysis of the target GhSADs, we employed the transient expression of these *GhSAD* genes in tobacco (*Nicotiana benthamiana*) leaf tissues and the heterologous expression in yeast (*Saccharomyces cerevisiae*) mutant *BY4389* lacking the synthesis of unsaturated fatty acids, followed by detecting fatty acid profiles in the target samples. In the whole, the present data evidence that GhA-SAD6 and GhD-SAD8 are responsible for making palmitoleic acid in developing cotton endosperm, providing novel insight to understand oil and ω-7 FA biosynthesis in plant seeds and scientific reference for lipid metabolic engineering in commercial oilseeds.

## Materials and Methods

### Plant Materials

Upland cotton (*G. hirsutum* cv. Zhong Mian 21) was planted in the experimental station at Shanxi Agricultural University, Taigu, China. Roots, stems and leaves were sampled from 6-week-old seedlings. Whole flower at 0 day after flowering (DAF) and developing ovules were sampled at 5, 10, 15, 20, 25, 35, and 45 DAF. Then ovules at 15, 25, 35 and 45 DAF were dissected manually to separate the embryos from the endosperms. All samples were instantly frozen in liquid nitrogen and stored at -70°C for subsequent experiment. Tobacco (*N. benthamiana*) was grown in an artificial incubator keeping at 26°C with 60% relative humidity and 14 h light/10 h dark photoperiod (Zhang et al., 2014).

### Gene Identification, Functional Domain, Three-Dimensional Model and Promoter Analysis

The conserved FA_desaturase_2 domain (PF03405) was retrieved from Pfam database^1^ and used as the query to perform BLAST against proteins of *G. hirsutum* from the CottonGen database^2^. The sequence features were identified by HMMER version 3.0 with default parameters, and their conserved motifs were further verified by SMART^3^ and CDD tool^4^. Only the sequence containing conserved FA_desaturase_2 domain was employed for further analysis. All candidate *GhSAD* genes were renamed according to their chromosomal distributions, denoting *GhA-SAD1*∼*GhA-SAD9* and *GhD-SAD1*∼*GhD-SAD8*. Transcriptional data were downloaded from cotton research institute of Nanjing Agricultural University^5^.

For *cis*-element analysis, the extracted 2-kb upstream sequences from the initiation codon of all *GhSADs* were searched in the PlantCARE database^6^. The data of AtSAD proteins were downloaded from TAIR database^7^. Castor SAD sequences and SAD in cat’s claw (Muc-PAD) were downloaded from NCBI^8^. To investigate the key amino acid residues that influence the catalytic properties of SAD enzyme, amino acid sequence comparisons of all GhSAD proteins and the reference protein sequences (RcSAD1, AtFAB2, AtAAD3, AtAAD2, and Muc-PAD) were conducted. Then, three-dimensional structures of GhSADs were modeled using Swiss-model^9^, with the crystal structure of RcSAD1 (NP_001310659.1) as the templet. In addition, the key amino acids and ligands in catalytic activity center were marked using Discovery Studio 4.1 software, with the default parameters.

### RNA Isolation and Real-Time Quantitative PCR

EASYspin Plus Plant RNA Kit (Aidlab, Beijing, China) was used for the isolation of total RNA from all sample types. First-strand cDNA was reversely transcripted using PrimeScript^TM^ II 1st Strand cDNA Synthesis Kit (Takara, Kyoto, Japan) according to the manufacturer’s instruction. Gene-specific primers used were listed in Supplementary Table S1. The experiment was carried out on a Bio-Rad CFX96 Real-Time PCR system (Bio-Rad, United States) with three replicates. Following reaction conditions were applied: 95°C for 10 min; 40 cycles of 95°C for 15 s, 60°C for 1 min; 72°C for 20 s. The melting curve was analyzed after the final cycle, ramping from 65 to 95°C with increment 0.5°C/5 s. The normalized relative expression was calculated using the 2^-ΔΔCq^ method (Livak and Schmittgen, 2001). Cotton *EF-1α* (DQ174251) and *histone 3* (AF024716.1) genes were employed as the internal references (Jiang et al., 2012; Shang et al., 2015).

### Construction of Plant and Yeast Expression Vectors of the *GhSAD* Members

To test their substrate preferences, the full-length open reading frames (ORFs) of four representative *GhSAD* genes (*GhA-SAD5, GhA-SAD6, GhA-SAD7*, and *GhD-SAD8*) were amplified by PCR with oligonucleotides (Supplementary Table S2) and then cloned into pCAMBIA1303 vector under the drive of cauliflower mosaic virus (CaMV) 35S promoter. The resulting vectors were transferred into *Agrobacterium* strain of GV3101 using the freeze-thaw method for further transient expression in tobacco leaves by infiltration. The empty vector pCAMBIA1303 was used as blank control. For functional analysis using yeast expression system, the target *GhSAD* genes with optimized codons were then cloned into the yeast expression vector pYES2 under the control of the *GAL1* promoter. Additionally, *AtFAB2* and *AtAAD3* from *A. thaliana* were cloned according to the published sequences and inserted into pYES2 as positive controls. Empty vector of pYES2 was set as blank control. Related primers were listed in Supplementary Table S2.

### Transient Expression Assays in *N. benthamiana* Leaves

The constructed plant expression vectors were transferred into *Agrobacterium tumefaciens* strain GV3101. Agroinfiltration was employed for transient expression of the target genes in tobacco leaves. Briefly, the bacteria bearing the vector were cultured in LB medium and incubated at 28°C overnight with 250 rpm. Bacterial cultures were then centrifuged at 8,000 rpm for 3 min and resuspended into fresh infiltration medium supplemented with acetosyringone, MgSO_4_ and MES to an optical density of 0.5 at 600 nm. Agroinfiltration was performed using a needle-free syringe in the leaves of six-week-old *N. benthamiana* plants. The treated leaves were harvested 5 days later, and then ground up into powder for the extraction of total lipids.

### Yeast Culture and Transformation

The mutant yeast *S. cerevisiae* strain *BY4389* (*MATa ole1Δ::LEU2 ura3-52 his4*) that cannot synthesize unsaturated fatty acids was purchased from Osaka University, Japan (Xue et al., 2016). Before transformation, the cells were cultivated at 28°C in modified yeast-extract peptone dextrose (YPD) medium (0.01% sodium oleate, 1% yeast extract, 2% peptone and 2% glucose) with shaking (200 rpm). When the concentration was at OD600≈0.5, the cells were harvested by centrifugation, washed three times with precooling sterilized water. Then, the cells were resuspended in 100 mM LiAc solution. After removing LiAc, we got the competent cells of yeast with high transformation efficiency. The constructed yeast expression vector and empty pYES2 were separately transformed into *S. cerevisiae* mutant strain *BY4389* using Yeast Transformation Kit (Coolaber, Beijing, China). Transformed cells were selected on the modified YPD medium lacking uracil (SC-Ura). After cultured 3 days at 28°C, PCR confirmation was used to select positive clones. The correct transformed cells were then transferred in the corresponding selective medium to increase the yeast biomass for 24 h. For induction, glucose was replaced by galactose (2%, w/v) and sodium oleate was removed, and cells were further cultivated for another 72 h. At last, transformed cells were harvested and freeze-dried into powder to for fatty acid analysis.

### Lipid Extraction and Fatty Acid Methyl Ester Analysis

Total lipids from developing cotton embryo and endosperm (15, 25, 35 and 45 DAF), the wild and transgenic *N. benthamiana* leaves and yeast samples were extracted, and then converted into fatty acid methyl esters (FAMEs) as previously described (Bhattacharya et al., 2015). Briefly, FAMEs were transmethylated using 2 mL of methanol that contained 2.5% sulfuric acid (v/v) and then incubated at 85°C for 2 h. For quantification of the FAs, an appropriate amount of C17:0 FAME (Sigma) was added as internal standard. FAMEs were analyzed by Agilent gas chromatograph equipped with a flame ionization detector and a capillary column (HP-88 100 m × 0.25 mm × 0.2 μm). Nitrogen was used as carrier gas with a flow rate of 2.64 ml/min. 1 μL of the sample was injected into the column with three replicates. The injection and detector temperatures were both set as 250°C. The gas chromatograph oven was programmed at an initial temperature of 140°C for 5 min, and then increased up to 250°C at a rate of 15°C/min. Fatty acid component was estimated according to the retention time of fatty acid standard and data was collected by peak area normalization with C17:0 internal standard.

## Results

### Differential Distribution of Fatty Acids Between Embryos and Endosperms in Developing Cottonseeds, Especially for Palmitoleic Acid

**FIGURE 1:**
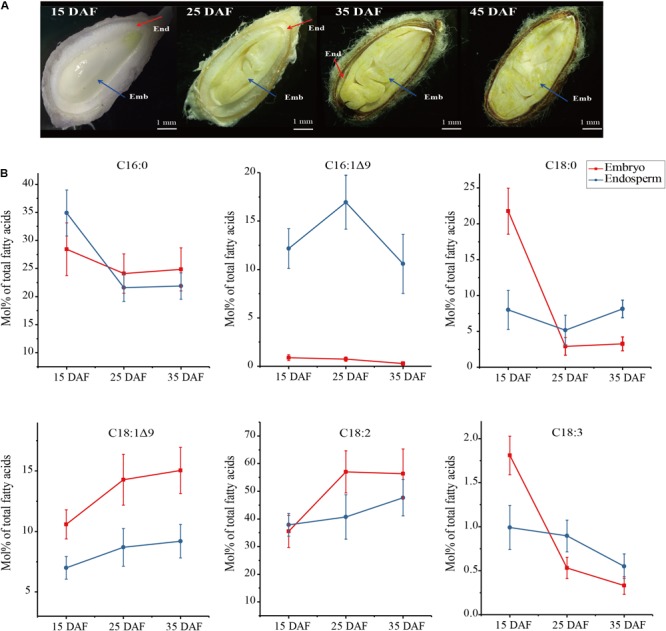
Four development stages of cotton seeds **(A)** and fatty acid profiles in developing embryo and endosperm **(B)**. Fatty acid content (Mol%) was presented as a percentage of all fatty acids in embryo and endosperm, respectively. Values are the mean ± SE of six biological duplicates.

Angiosperm seed formation is characterized by the double fertilization of the embryo sac. During this process, one sperm cell fertilizes the egg cell to produce a zygote, which develops into an embryo, whereas the second sperm cell targets the central cell polar nuclei, leading to the endosperm formation. Both the diploid embryo and the triploid endosperm are enclosed by the maternally derived ovular integument layers that form seed coat. As a typical exalbuminous seed, cotton seed has no endosperm left at mature stage despite abundant endosperms surrounding the embryo are generated during the early seed development, providing nutrition for the enlarging embryo as it develops (Figure 1A).

To investigate whether fatty acid profiles are different between embryos and endosperms during development, we harvested embryo and endosperm samples, respectively, at four stages of seed development denoted as 15 DAF (days after flowering), 25, 35, and 45 DAF (Figure 1A). At the early stage of 15 DAF, the endosperm forms a thick milky layer surrounding the embryo. During seed development and maturation, the endosperm volume is gradually reduced into a thin layer closely to seed shell (25–35 DAF), accompanied by the embryo growing. At the end of seed development (45 DAF), the endosperm is disappeared in cotton seed.

The examination of FA contents in developing cotton seeds (Figure 1B) showed that FA profiles were significant different between endosperm and embryo. The most difference is that 16:1Δ^9^, a kind of ω-7 FAs, was largely accumulated in endosperm with the peak at 25 DAF (16.95 Mol% of total FAs), but only trace level (<1 Mol%) in embryo. However, content of 18:1Δ^9^, a kind of ω-9 FAs, was higher in embryo than in endosperm by 1.2–1.7 folds, with the maximum level (15.04 Mol%) in embryo at 35 DAF. Palmitic acid (16:0) was the major FA in endosperm at 15 DAF, and it reduced gradually during seed development, with lower level in endosperm than in embryo from 25 to 35 DAF. Linoleic acid (18:2Δ^9,12^), the major FA in embryo, is increased up to ∼60% in embryo at 25 DAF, with lower level in developing endosperm. The contents of 18:0 and 18:3Δ^9,12,15^ were not significant between embryo and endosperm from 25 to 35 DAF despite these two fatty acid contents were higher in embryo than in endosperm at 15 DAF of the seed development. Notably, abundant palmitoleic acid (16:1Δ^9^) were found to be selectively accumulated in developing endosperm, suggesting that some unknown mechanism may control this differential accumulation of palmitoleic acid between the endosperm and embryo.

### Identification of Seventeen Stearoyl-ACP Δ^9^ Desaturases (GhSADs) and Six Putative 16:0-ACP Specific GhSADs From Upland Cotton

As described in the introduction, some members (if not all) of SAD family can catalyze palmitic acid (16:0)-ACP to form palmitoleic acid (16:1Δ^9^)-ACP in plant seeds. The preferred accumulation of 16:1Δ^9^ in the developing cotton endosperm observed above indicates that SAD enzyme working in endosperm may have high substrate specificity for palmitic acid (16:0). To investigate such SAD members with high 16:0-ACP specificity, we performed a genome-wide characterization of SAD family members in upland cotton (*G. hirsutum*) genome. Total of 17 putative GhSADs were identified according to the conserved domain and their integrity. These predicted GhSADs were designated as *GhA-SAD1*∼*GhA-SAD9* and *GhD-SAD1*∼*GhD-SAD8* based on chromosome number of A- or D-genome where they located (Supplementary Table S3).

The known reports have established that RcSAD1 from castor (*Ricinus communis*) (Lindqvist et al., 1996) and AtFAB2 from Arabidopsis (Lightner et al., 1994) are characterized as the 18:0-ACP specific SADs while AtAAD2 and AtAAD3 from Arabidopsis (Troncoso-Ponce et al., 2016) as well as Muc-PAD from cat’s claw (*Macfadyena unguis-cati*) (Cahoon et al., 1998) are identified as the 16:0-ACP specific enzymes. Moreover, the substrate specificity of these enzymes is determined by a group of eight amino acid residues comprising the region of the catalytic sites (also referred as the lower portion of the hydrophobic binding pocket for FA). To identify which GhSAD may be the candidate of 16:0-ACP specific enzyme, these well-characterized SADs were used as the references to perform *in silico* analyses of 17 GhSAD sequences including sequence comparisons and modeling of 3D protein structures (Arnold et al., 2006; Bordoli et al., 2009).

**FIGURE 2:**
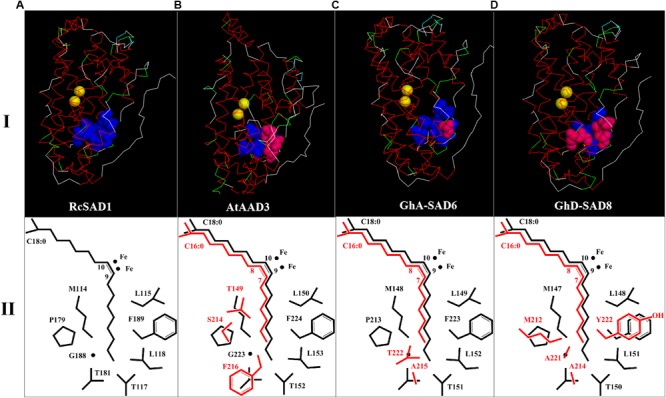
3D structure models of GhSAD protein monomers (**C,D**, upper panel I) and C18:0/C16:0-ACP chains as well as side chains of key amino acids close to catalytic center of diiron ions (lower panel II). All 3D models were predicted on Swiss-model (https://swissmodel.expasy.org/) with RcSAD1 (NP_001310659.1) and AtAAD3 as temples **(A,B)**. Files of 3D structure of GhSADs were showed using Discovery Studio 4.1 software. Yellow bolls in upper panel I refer to diiron ions. Blue bolls/spheres refer to common amino acids as same as RcSAD1 while pink bolls refer to the varied amino acids. The fatty-acyl chains and side chains of amino acids were drawn by ChemDraw software (lower panel II). Black color represents C18:0-ACP and its common amino acids. Red indicates C16:0-ACP and varied amino acids.

Table 1 summarized the comparison of the eight amino acid (AA) residues lining the lower portion of the hydrophobic binding pocket for FA in those enzymes tested. Seventeen GhSADs were clearly divided into two groups. Group 1 with ten GhSAD members had the identical AA residues in the conserved functional domains as RcSAD1 and AtFAB2 (Table 1), which are ω-9 producing SAD isoforms. Group 2 consisting of GhA-SAD5, 6, 7, and GhD-SAD4, 5, 7, 8 were gathered together with 16:0-ACP specific Muc-PAD, AtAAD2 and AtAAD3, although divergent residues existed in the eight-AA domain of those enzymes. The 3D model revealed that the eight AA residues were located near the bottom of the substrate-binding channel close to diiron center of the enzymes examined (Figure 2I). The identical eight-AA domain in the 18:0-ACP specific enzymes is predicted to shape a deep/long substrate cavity (pocket/channel) to accommodate 18:0-ACP substrate (Figure 2A). However, the divergent eight-AA domain in the 16:0-ACP specific enzymes is predicted to make the substrate channel shorter, which is more adaptable for 16:0-ACP substrate (Figure 2B,C,D).

**Table 1:** Comparison of the eight amino acid residues comprising the region of the catalytic sites of GhSADs.

*Ricinus communis*	*Arabidopsis thaliana*	*Gossypium hirsutum*									
	
RcSAD1	AtFAB2	GhA-SAD1	GhA-SAD2	GhA-SAD3	GhA-SAD4	GhA-SAD8	GhA-SAD9	GhD-SAD1	GhD-SAD2	GhD-SAD3	GhD-SAD6
**Group 1:ω-9 producing isoforms**											
L118	L156	L141	L150	L140	L151	L150	L150	L150	L141	L151	L150
F189	F227	F212	F211	F211	F212	F221	F221	F221	F212	F222	F221
L115	L153	I138	L147	I137	L148	L147	L147	L147	I138	L149	L147
T117	T155	T140	T149	T139	T150	T149	T149	T149	T140	T150	T149
G188	G226	G211	G210	G210	G211	G220	G220	G220	G211	G221	G220
T181	T219	T204	T213	T203	T214	T213	T213	T213	T204	T214	T213
P179	P217	P202	P211	P201	P212	P211	P211	P211	P202	P212	P211
M114	M152	M137	M146	M136	M147	M146	M146	M146	M137	M148	M146

***Arabidopsis thaliana***		***Dauricum unguis-cati***		***Gossypium hirsutum***						
**AtAAD3**	**AtAAD2**	**Muc-PAD**		**GhD-SAD4**	**GhA-SAD5**	**GhD-SAD5**	**GhA-SAD6**	**GhA-SAD7**	**GhD-SAD7**	**GhD-SAD8**
**ω-7 producing isoforms**				**Group 2: ω-7 producing isoforms?**						

L153	L163		Y1W152	L151	L31	L150	L152	P1M148	P1M148	L151
F224	F234		F223	F222	F102	F221	F223	P1A219	P1 A219	P1Y222
L150	L160		L149	L149	L28	L147	L149	L145	L145	L148
T152	T162		T151	T150	P1R30	P1R149	T151	T147	T147	T150
G223	G233		G222	G221	P1T101	P1T220	P1T222	G218	G218	P1A221
Y1F216	Y1F226		T215	T214	P1I94	P1I213	P1A215	P1S211	P1S211	P1A214
Y1S214	Y1S224		P213	P212	P1V192	P1V211	P1P213	P1S209	P1S209	P1M212
Y1T149	Y1T159		M148	P1I148	P1I27	M146	M148	P1L144	P1L144	M147

*Sequences were conducted by GeneDoc software using RcSAD1, AtFAB2, AtAAD3 as well as Muc-PAD as temples. The GenBank or TAIR accession numbers of the temple sequences listed as the follows: RcSAD1 (NP_001310659.1) from *R. communis*; AtFAB2 (At2g43710), AtAAD2 (At3g02610), and AtAAD3 (At5g16230) from *A. thaliana*; Muc-PAD (AFV61670.1) from *D. unguis-cati*. Orange and pink color represent varied amino acids residues in the domain of 16:0-ACP specific isoforms and the putative 16:0-ACP specific GhSADs, respectively, compared to the 18:0-ACP specific isoforms characterized.*

To further test whether these GhSADs in Group 2 are the candidate enzymes responsible for 16:1Δ^9^ biosynthesis in developing cotton endosperm, *cis*-elements analyses were conducted on the promoter sequences of the corresponding genes. The promoters of *GhA-SAD5, GhA-SAD6, GhA-SAD7, GhD-SAD5, GhD-SAD7* and *GhD-SAD8* contained multiple *skn-1* motifs with the core sequences of GTCAT which is necessary for gene expression in endosperm (Zhang et al., 2013). In addition, *GhD-SAD5* and *GhD-SAD8* have another GCN4 motif (the core sequences is TGAGTCA) involved in gene expression in endosperm (Supplementary Table S4) (Song et al., 2012). However, the promoter of *GhD-SAD4* lacked such *cis*-element. Overall, sequence analysis indicate that GhA-SAD5, 6, 7, and GhD-SAD5, 7, 8 may be the putative 16:0-ACP specific SADs responsible for palmitoleic acid biosynthesis in developing cotton endosperm.

### Expression Patterns of *GhSAD* Genes in Different Cotton Tissues

**FIGURE 3:**
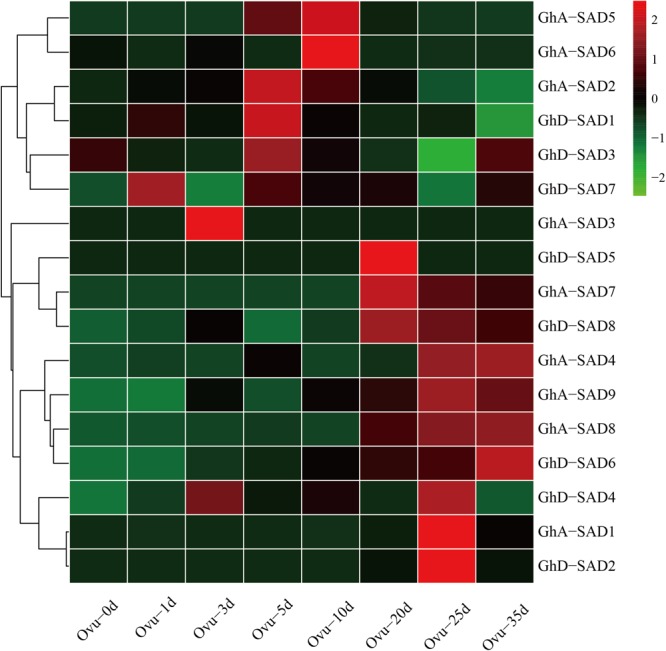
Transcriptional expression patterns of 16 GhSAD family members during developing cotton ovule. The RNA-seq data used here were derived from NCBI database. The developing ovule stages were listed as 0d, 1d, 3d, 5d, 10d, 20d, 25d, and 35d after flowering (DAF) in developing cotton ovules.

If any of GhSADs identified above functions in endosperm palmitoleic acid production, it must be expressed in developing cotton endosperm. To determine this, we firstly examined their transcript abundance during developing ovules using cotton RNA-seq profiles publically available (see text footnote 5). As shown in Figure 3, these 17 *GhSADs* exhibited different expression patterns in developing ovules. High expressions of *GhD-SAD7, GhA-SAD3*, and *GhD-SAD4*, occurred at the early stages of ovules. *GhA-SAD2, GhD-SAD1* and *GhD-SAD3* abundantly appeared at 5 DAF. Seven *GhSAD* (*GhA-SAD5, GhA-SAD6, GhD-SAD5, GhA-SAD-7, GhD-SAD8, GhA-SAD1*, and *GhD-SAD2*) transcripts greatly accumulated at the middle stage of ovules (from 10 DAF to 25 DAF), while *GhA-SAD4* and *GhD-SAD6* largely expressed at the late stage of ovules (35 DAF).

**FIGURE 4:**
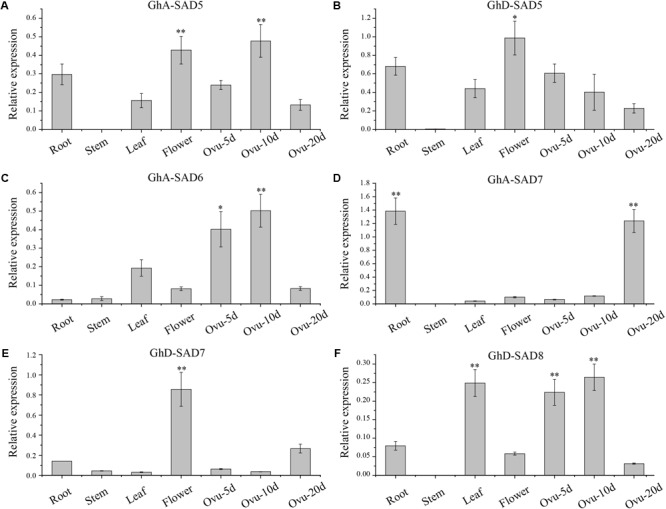
Deduced *GhSAD* expression patterns of *GhA-SAD5* **(A)**, *GhD-SAD5* **(B)**, *GhA-SAD6* **(C)**, *GhA-SAD7* **(D)**, *GhD-SAD7* **(E)**, and *GhD-SAD8* **(F)** in various cotton tissues. Bar charts show the relative expression levels of *GhSAD* genes normalized to that of *Histone3* and *EF-1*α measured by qRT-PCR. The analysis was performed with three biological samples for each tissue. Ovu-5d, 10d and 20d represent 5, 10, and 20 DAF of developing cottonseed ovules. The method of 2^-ΔΔcq^ was used in this analysis. “^∗^” represents statistically significant difference values of *P* < 0.05 and “^∗∗^” for *P* < 0.01 based on two-tailed Student’s *t*-tests.

To further verify the expression patterns of these *GhSADs*, we employed qRT-PCR to investigate their mRNA levels in various cotton tissues (Figure 4). Six *GhSADs* with high expressions were selected for this verification. Specific primers for qRT-PCR were designed at 3′ untranslated regions of the *GhSADs* because of their highly conserved ORF sequences (Supplementary Table S1). As shown in Figure 4, *GhA-SAD5, GhA-SAD6*, and *GhD-SAD8* were highly expressed in the early middle developing ovules (5 and 10 DAF) while *GhA-SAD7* enriched at the late developing stage (20 DAF). *GhA-SAD7* also had the highest expression level in root compared to other tissues, indicating that GhA-SAD7 may function importantly in root. For flower tissue, both *GhD-SAD5* and *GhD-SAD7* had abundant expression levels, implying that GhD-SAD5 and GhD-SAD7 play roles in flower development. It is known that the fastest period of oil/FA biosynthesis in cotton endosperm was the middle stage (10–15 DAF) of the developing ovule. Combination of RNA-seq and qRT-PCR data suggests that GhA-SAD6, GhD-SAD8, GhA-SAD5, and GhA-SAD7 may play roles in FA/oil biosynthesis in developing cotton endosperm.

### *GhA-SAD6* and *GhA-SAD8* Mostly Expressed in Developing Endosperm, Positively Correlating With Palmitoleic Acid Accumulation in Cotton Seeds

To explore whether those *GhSADs* observed above are associated with 16:1Δ^9^ production in endosperm, developing cotton seeds at 15, 25, 35, and 45 DAF were then dissected and the two fractions, embryo and endosperm, were separately obtained to examine FA profiles and the expression patterns of the four *GhSAD* genes (*GhA-SAD5, GhA-SAD6, GhA-SAD7*, and *GhD-SAD8*) were selected according to the above characterization.

**FIGURE 5:**
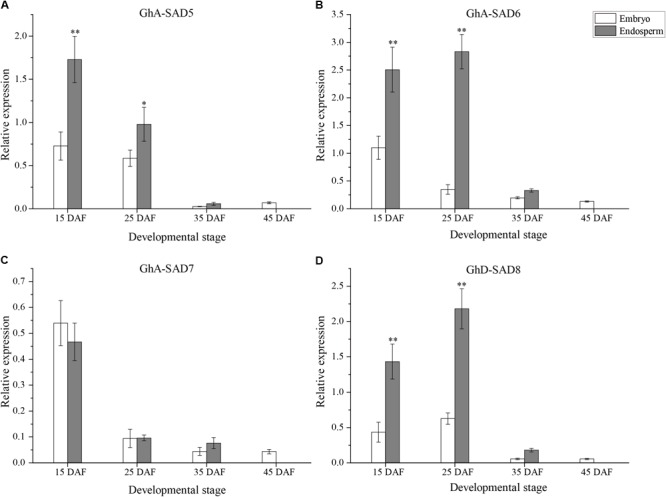
Expression patterns of *GhA-SAD5* **(A)**, *GhA-SAD6* **(B)**, *GhA-SAD7* **(C)**, and *GhD-SAD8* **(D)** in developing cotton embryos and endosperms at 15, 25, 35, and 45 DAF. The same experimental and statistical methods are performed as in Figure 4.

As shown in Figure 5, the four *GhSAD* genes exhibited differential expression patterns between embryo and endosperm of cotton seeds. The mRNA levels of *GhA-SAD5, GhA-SAD6*, and *GhD-SAD8* were significantly high in the endosperm but very low in the embryo during seed development from 15 to 25 DAF, a period of palmitoleic acid level increased rapidly in the endosperm (Figure 5A,B,D). However, *GhA-SAD7* expression was no obvious difference between embryo and endosperm (Figure 5C). Only a trace level of the four gene transcripts was detected in the embryo at the stage of 45 DAF. By this time, no endosperm was left in the seed. In addition, all these four genes had extremely low expression levels in cotton embryos and endosperms at 35 DAF compared to the two early stages (15 and 25 DAF). All these data suggest that GhA-SAD6 and GhD-SAD8 may be the main players for fast oil and palmitoleic acid accumulation in developing cotton endosperm although *GhA-SAD5* has a higher expression in endosperm at 15 DAF.

**FIGURE 6:**
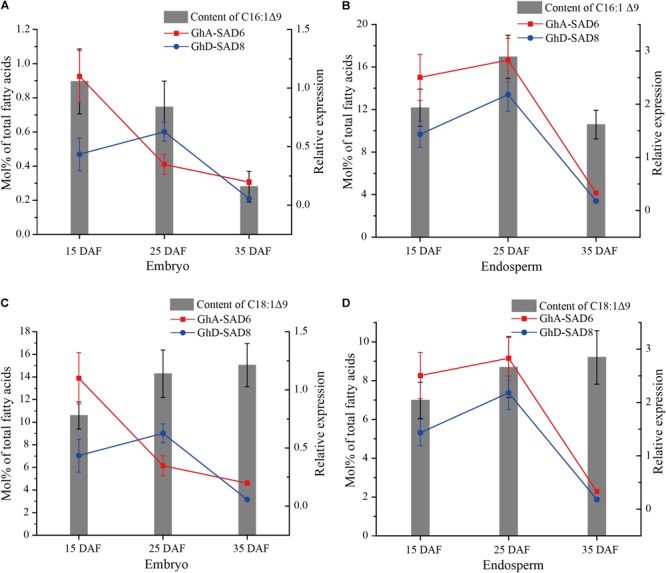
The correlation of the expression of *GhA-SAD6* and *GhD-SAD8* with molar percentage of C16:1Δ^9^ or C18:1Δ^9^ in developing cotton embryos and endosperms. Columns showed fatty acid content (Mol% of total fatty acids) and lines for relative expression (Red for *GhA-SAD6*, blue for *GhD-SAD8*). **(A,C)** represented the correlation between the fatty acid content (C16:1Δ^9^ or C18:1Δ^9^) and relative expression levels of *GhA-SAD6* or *GhD-SAD8* in developing embryos, respectively. **(B,D)** were the correlation of C16:1Δ^9^ or C18:1Δ^9^ content with *GhA-SAD6* or *GhD-SAD8* in developing endosperms, respectively. Six biological replicates were analyzed in each tissue.

To further confirm the association between *GhA-SAD6* and *GhD-SAD8* expressions and palmitoleic acid level in the endosperm of developing cotton seeds, two set of data, MUFA (16:1Δ^9^ and 18:1Δ^9^) contents and expression levels of *GhA-SAD6* and *GhD-SAD8* were integrated into one figure (Figure 6). In the embryo, neither 16:1Δ^9^ nor 18:1Δ^9^ content change was obviously correlated with the expression level of either *GhA-SAD6* or *GhD-SAD8* gene (Figure 6A,C). In the endosperm, no correlation was also detected between 18:1Δ^9^ content and the two gene expressions (Figure 6D). However, a significantly positive correlation was detected between 16:1Δ^9^ content and *GhA-SAD6* or *GhD-SAD8* expression in the endosperm fractions (Figure 6B). Moreover, linear regression analysis also showed that the level of palmitoleic acid in the endosperm is positively correlated with the expression of *GhA-SAD6* (*r* = 0.752) and *GhD-SAD8* (*r* = 0.886), respectively, again indicating that GhA-SAD6 and GhD-SAD8 may be the key contributors for biosynthesis and accumulation of palmitoleic acid in cotton endosperm.

### GhA-SAD6 and GhD-SAD8 Boosted Accumulation of Palmitoleic Acid When Transient Expression in *N. benthamiana* Leaves

**FIGURE 7:**
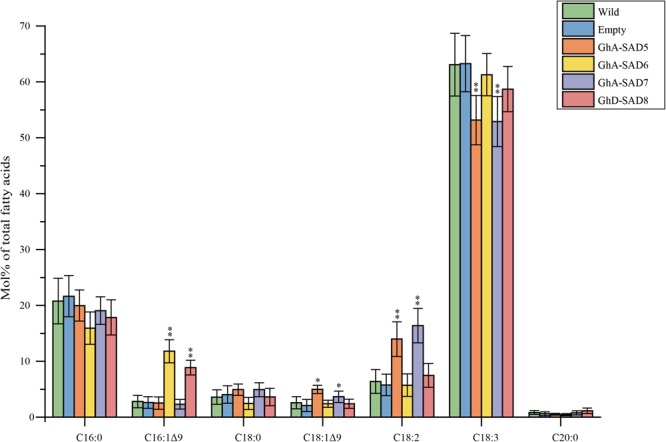
Fatty acid composition in transgenic *N. benthamiana* leaves of expressing *GhA-SAD5, GhA-SAD6, GhA-SAD7*, and *GhD-SAD8*, respectively. Fatty acids were analyzed by GC and the data are means of Mol% ± SE with six biological replicates. “^∗^” and “^∗∗^” represent statistically significant difference values from Wt (or empty) of *P* < 0.05 and *P* < 0.01 based on two-tailed Student’s *t*-tests, respectively. Empty vector of pCAMBIA1303 was used as negative control. Data are showed as mean ± SE (*n* = 6).

In order to identify the roles of GhA-SAD6 and GhD-SAD8 for biosynthesis of palmitoleic acid (16:1Δ^9^), we transiently overexpressed them separately in leaves of *N. benthamiana* by *Agrobacterium*-mediated infiltrating. *GhA-SAD5* and *GhA-SAD7* were also transiently expressed, respectively, in tobacco leaves for comparison. The transformed leaves were harvested at 5 days after infiltration, and used for lipid extraction. FA analysis by GC (Figure 7) revealed that compared to the wild-type and empty-vector controls, either *GhA-SAD6* or *GhD-SAD8* overexpression led to a significant increase of palmitoleic acid by at least 4–5 folds. However, *GhA-SAD5* or *GhA-SAD7* expression alone resulted in a slight enhance of oleic acid (18:1Δ^9^) by 2 folds around, with no effects on palmitoleic acid biosynthesis. These results indicate that GhA-SAD6 and GhD-SAD8 are specific for the ectopic enrichment of palmitoleic acid (e.g., to catalyze 16:0-ACP into 16:1Δ^9^-ACP) in the leaves whereas GhA-SAD5 and GhA-SAD7 are specific for oleic acid biosynthesis (e.g., to catalyze 18:0-ACP into 18:1Δ^9^-ACP). Other changes of FA profiles were the increase of linoleic acid (18:2Δ^9,12^) but reduction of linolenic acid (18:3Δ^9,12,15^) in the *GhA-SAD5*- or *GhA-SAD7*-expressed leaves. Such change in polyunsaturated fatty acid levels maybe due to the activity changes of the endogenous NbFAD2 and NbFAD3 enzymes in tobacco leaves.

### GhA-SAD6 and GhD-SAD8 Accounted for Palmitoleic Acid Production When Expressed in Yeast Mutant *BY4389*

To further confirm the function of GhA-SAD6 and GhD-SAD8 for biosynthesis of palmitoleic acid (16:1Δ^9^), we overexpressed the four *GhSAD* genes (*GhA-SAD5/6/7* and *GhD-SAD8*), respectively, in yeast mutant *BY4389* disable in synthesis of unsaturated fatty acids. For this genetic complementary assays, Arabidopsis AtFAB2 (18:0-ACP specific) and AtAAD3 (16:0-ACP specific) were employed as the positive controls, and an empty pYES2 vector was used as the negative control. FA composition was examined in the transformed *BY4389* strains.

**FIGURE 8:**
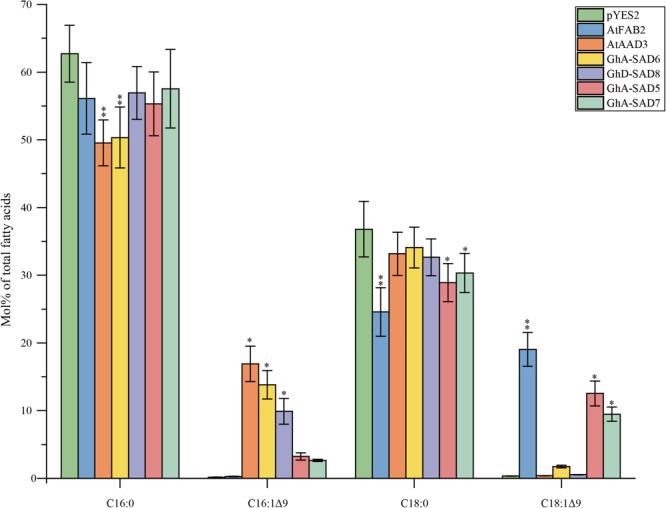
Fatty acid composition in mutant yeasts of *BY4389* of expressing *GhA-SAD5, GhA-SAD6, GhA-SAD7*, and *GhD-SAD8*, respectively. Fatty acids were analyzed by GC and the data are means of Mol% ± SE with six biological replicates. “^∗^” and “^∗∗^” represent statistically significant difference values from Wt (or empty) of *P* < 0.05 and *P* < 0.01 based on two-tailed Student’s *t*-tests, respectively. AtFAB2 and AtAAAD3 were used as positive control and empty vector of pYES2 as negative control. Data are showed as mean ± SE (*n* = 6).

Notably, all transformed yeast mutants except for empty pYES2 vector restored their desaturase function, and thus survived in SC-Ura medium without any unsaturated fatty acid. FA profiles detected (Figure 8) showed that the transgenic yeasts synthesized new but unbalanced-level unsaturated fatty acids (UFAs). As expected, *AtAAD3*-transgenic yeast generated 16.9 Mol% of C16:1Δ^9^ while *AtFAB2*-transgenics produced 19.03 Mol% of C18:1Δ^9^. Like in the *AtAAD3*-transgenics, both *GhA-SAD6*- and *GhD-SAD8-*expressed yeast accumulated high levels of C16:1Δ^9^ by 13.83 and 9.88 Mol%, respectively, but almost no detectable of C18:1Δ^9^. In contrast, the yeast mutants harboring *GhA-SAD5* or *GhA-SAD7* produced large amount of C18:1Δ^9^ by approximately 10 Mol%, with <3.5 Mol% of C16:1Δ^9^, which was similar to FA profiles in the AtFAB2-transgenic yeast. Overall, the present data obtained by *in vivo* assays using yeast mutant transformation again revealed that GhA-SAD6 and GhD-SAD8 both had palmitoyl-ACP substrate specificity, with the former stronger. Whereas, GhA-SAD5 and GhA-SAD7 showed to prefer stearoyl-ACP substrate over palmitoyl-ACP despite of low enzymatic activities.

## Discussion

### Enrichment of ω-7 FAs in Developing Cotton Seeds May Benefit the Embryo Development

For the albuminous seeds such as cereal grains and castor beans, the embryo is embedded in the endosperm which persists to the mature seed stage as a storage tissue supplying nutrients for the seedling that will grow from the embryo (Huh et al., 2007). However, for exalbuminous seeds such as soybean, peanut and cotton, the endosperm is absorbed by the embryo during seed development, and the cotyledons become filled with stored food (Yan et al., 2014). And thus, no endosperm is left in maturity seeds. A differential partitioning of reserve compounds between different tissues in Arabidopsis seeds was examined, with a dramatically increased accumulation of the economically important ω-7 FAs in the endosperm, but not in the embryo (Penfield et al., 2004; Barthole et al., 2014). Here, we demonstrate that palmitoleic acid (16:1Δ^9^) is enriched in developing cotton endosperm but not in the embryo. This ω-7 FA accumulation is different in Arabidopsis and rapeseed where this unusual fatty acid is highly concentrated in a single cell layer (aleurone) of the residual endosperm surrounding the embryo in the mature seed, with the major species being *cis*-vaccenic acid (18:1Δ^11^) and paullinic acid (20:1Δ^13^) instead of palmitoleic acid (16:1Δ^9^) (Li et al., 2006). Such difference suggests that there may be different temporal-spatial regulation mechanisms in different plant seeds. And it also raises the question of what is the biological function of these ω-7 FAs enriched in the developing endosperm or the residual endosperm.

In Arabidopsis, transcriptional factors MYB115 and MYB118 coordinately upregulate the transcription of AtAAD2 and AtAAD3 which catalyze the synthesis of ω-7 FAs, leading to high levels of ω-7 FA accumulation exclusively in the endosperm of early maturing seeds (Bryant et al., 2016; Troncoso-Ponce et al., 2016). Arabidopsis double mutant of *MYB115* and *MYB118* resulted in significant decrease of ω-7 FAs in the endosperm, concurrently with a slight delay in the elongation and enlargement of the embryo associated with a moderate decrease of mature seed dry weight. This indicates that ω-7 FAs may function for seed development. However, seeds from the double mutant of *AtAAD3* and *AtAAD2* appear normal and can germinate readily under standard growth conditions. Even so, function of ω-7 FAs for seed germination cannot be excluded because the low level of remained ω-7 FAs in the endosperm of this double mutant may impair the examination of possible phenotypes.

To clarify the physiological roles of ω-7 FAs enriched in developing cotton endosperm, we conducted an *in vitro* cultivation of young embryos isolated from early developing seeds (data not shown). Addition of palmitoleic acid into the medium promoted the embryo development, with the embryo growing more health compared to the embryo cultured in the medium without this ω-7 FA. Moreover, this beneficial effect of ω-7 FA addition exhibited much stronger under cold stress. This demonstrates that ω-7 FA accumulation in the developing endosperm may function importantly for cotton seed development. Further lab work such as CRISPR-Cas9 mediated 16:0-ACP specific GhSAD mutation in developing cotton endosperm is under way to investigate the biological functions of ω-7 FA, particularly for seed development and germination.

### Members of Cotton GhSAD Family May Function Differentially, With GhA-SAD6 and GhD-SAD8 as the Major Players for Palmitoleic Acid Enrichment in Developing Cotton Endosperm

Generally, soluble acyl-ACP Δ^9^ desaturases (AAD) catalyze the formation of first unsaturated fatty acid in FA biosynthesis pathway in plant plastid. As a polygenic family, AAD contains various isoforms. For example, multiple members of AAD family were identified in several plant genomes, with 7 members in Arabidopsis (Kachroo et al., 2007), 8 members in cacao (*Theobroma cacao*) (Zhang Y. et al., 2015) and 3 members in olive (*Olea europaea*) (Parvini et al., 2016), respectively. Moreover, different AAD isoforms showed functional diversity and distinct expression patterns. Among 7 AtSAD members, SSI2/FAB is the predominant and the best characterized isoform, with expression in all seed tissues and 18:0-ACP Δ^9^ desaturation (Lightner et al., 1994) despite it was also active on 16:0-ACP (Kachroo et al., 2007). Microarray analysis by Le et al. (2010) showed that *AtAAD2* expression was mainly in the peripheral endosperm, whereas *AtAAD3* largely expressed in the chalazal region of the endosperm (Le et al., 2010; Bryant et al., 2016). Further analysis on the single or double mutant of the genes by Bryant et al. (2016) evidenced that AtAAD2 and AtAAD3 function in 16:0-ACP Δ^9^ desaturation and contribute to the biosynthesis of ω-7 FAs specifically in the aleurone. Possibly, the accumulation of ω-7 FAs in rapeseed aleurone plus seed coat results from the homologs of AtAAD3 since rapeseed has the relatively close phylogenetic relationship with Arabidopsis (Bryant et al., 2016). Although such findings are obtained from the characterization of the multigene family of AAD, the functions of most isoforms of this family remain to be elucidated.

Previous study showed that total of 17 stearoyl-ACP Δ^9^ desaturases (SAD) were identified in upland cotton genome, and their expression were developmentally regulated (Shang et al., 2017). However, no functional analysis of these 17 GhSADs is available, hence raising the question whether these GhSADs are redundant to the monounsaturated fatty acid formation or non-functional enzyme, or function separately from one another.

In the light of the present study, it demonstrates that the identical GhSAD family members were identified in upland cotton (Supplementary Table S3). Four GhSAD members including *GhA-SAD5, GhA-SAD6, GhA-SAD7* and *GhD-SAD8* showed higher expression levels at early stage of developing ovules than in other vegetative organs (Figure 4). Detailed expression analysis in dissection cottonseeds of different developmental stages revealed that the four genes above were highly expressed in early developing endosperm (15–25 DAF) (Figure 5), especially for *GhA-SAD6* and *GhD-SAD8* (Figure 5). However, the transcripts of *GhA-SAD2, GhA-SAD3*, and *GhD-SAD6* mostly accumulated in the embryo (data not shown). Such diverse spatiotemporal expression patterns indicate that these GhSADs tested may function differentially. In order to investigate their functions, particularly those GhSADs selectively expressed in developing cotton endosperm, we conduct *in vivo* functional assays including transient expression in tobacco leaves and heterologous expressing in yeast strain *BY4389* unable to synthesize unsaturated fatty acids. The overexpression of both *GhA-SAD6* and *GhD-SAD8* substantially increase palmitoleic acid (16:1Δ^9^) synthesis in leaves, accompanied by a concomitant decrease in palmitic acid (16:0) (Figure 7). Unlike *GhA-SAD6* and *GhD-SAD8*, the transient expression of either *GhA-SAD5* or *GhA-SAD7* slightly enhance oleic acid (18:1Δ^9^) in the leaves at the expense of stearic acid (18:0) (Figure 7). Similar results were also obtained by functional complementation assay in the yeast system (Figure 8). These reveal that in the developing cotton endosperm, GhA-SAD6 and GhD-SAD8 are responsible for the synthesis of palmitoleic acid (16:1Δ^9^) by 16:0-ACP desaturation while GhA-SAD5 and GhA-SAD7 are responsible for the synthesis of oleic acid (18:1Δ^9^) by 18:0-ACP desaturation. Together, our data demonstrate for the first time that the four GhSAD members (GhD-SAD8, GhA-SAD5, GhA-SAD6 and GhA-SAD7) are functional acyl-ACP Δ^9^ desaturase enzymes but with different substrate preferences. GhA-SAD6 and GhD-SAD8 can preferentially catalyze 16:0-ACP to 16:1Δ^9^-ACP, whereas GhA-SAD5 and GhA-SAD7 are able to selectively catalyze 18:0-ACP to 18:1Δ^9^-ACP although *in vitro* assay has not been conducted yet for identifying the substrate specificity of those GhSAD members. Evolutionally, the occurrence of multiple SAD members in upland cotton and other plant species tested likely provide diverse enzymes with selective expression so as to achieve fine spatiotemporal accumulation of different monounsaturated fatty acids as in cases of the seeds from cotton, Arabidopsis and rapeseed where fatty acid profiles were different between embryo and endosperm. Further functional characterization is required for every member of this SAD family. Nevertheless, our study brings new perspectives in this regard.

### Key Amino Acid Residues Lining the Bottom Part of the Substrate-Binding Channels of SADs Are Crucial Determinants of the Substrate Specificity of the Enzymes, but Not the Only Factor

As described above, plant SAD family composed of soluble plastidial proteins contains multiple members with differential expressions in different tissues and distinct substrate specificities. Some isoforms of the enzyme like castor RcSAD1 and Arabidopsis AtFAB2 show high preference for 18:0-ACP, yielding 18:1Δ^9^-ACP, while others such as cat’s claw Muc-PAD, Arabidopsis AtAAD2 and AtAAD3 exhibit strong activity on 16:0-ACP, producing 16:1Δ^9^-ACP. It have been reported that several Δ^9^ desaturases can recognize 14-, 16-, and 18-carbon chain lengths, and others perform desaturation with Δ^6^ and Δ^9^ regiospecificities (Whittle et al., 2005; Guy et al., 2007). In view of evolution, such diverse substrate specificities of SADs likely provide a competitive advantage for host plants to produce the required monounsaturated FAs for life activities including maintaining membrane fluidity and channeling into storage triacylglycerols (TAGs) in storage tissues (Shanklin and Cahoon, 1998), benefiting the evolution of novel metabolic diversity (Dyer et al., 2002).

Previous studies on the molecular mechanism underlying SAD substrate specificity were mostly conducted using the crystal structure of castor 18:0-ACP Δ^9^ desaturase (RcSAD1) as a model (Lindqvist et al., 1996; Cahoon et al., 1997), showing that a group of eight amino acid residues lining the bottom part of the substrate channels of SADs is crucial for determining the substrate specificity of the enzymes (Cahoon et al., 1998). For example, RcSAD1 could change its binding substrate for 16:0-ACP from the wild type with 18:0-ACP when two key residues (T117 and G188) were substituted by Arg and Leu. Moreover, the mutant RcSAD1 activity for 16:0-ACP was higher by 82 folds than the wild enzyme for 18:0-ACP (Whittle and Shanklin, 2001). The side chain sizes of these 8 amino acids influence the depth and width of the substrate channel of the enzyme (Cahoon et al., 1997). The substrate channel of the archetype Δ^9^ SAD is deep enough to accommodate 18:0-acyl chain, thus producing Δ^9^ monoenes (Figure 2A). However, the shorter substrate channel of the enzyme is more adapted to 16:0-acyl chain, forming a ω-7 FA (e.g., palmitoleic acid, 16:1Δ^9^) (Figure 2A–C). These eight amino acids appear to be well conserved among the SAD isoforms characterized so far in higher plants. The amino acid sequences of the most SAD isoforms are highly homology to that of the castor 18:0-ACP Δ^9^ desaturase (RcSAD1), implying that SADs share a common architecture able to accommodate different substrate binding models. Like in Arabidopsis, AtSADs share a high degree of amino acid sequence similarity and a common structural fold (Kachroo et al., 2007). Moreover, the three divergent residues (F226/216, S224/214, and T159/149) of the eight amino acids lining the bottom part of the substrate channels of AtAAD2 and AtAAD3 conferred their strong activity for 16:0-ACP, but not 18:0-ACP catalyzed by the SAD archetype (Bryant et al., 2016).

Base on such conservation, the corresponding amino acid residues in 17 cotton SADs were identified by a polypeptide sequence alignment of the eight amino acid residues lining the bottom part of the substrate channels of the GhSAD proteins using the typical 18:0-ACP specific SADs (RcSAD1 and AtFAB2) and the 16:0-ACP specific SADs (Muc-PAD, AtFAB2, AtAAD2, and AtAAD3) as references (Table 1). Interestingly, the eight amino acids are all identical for the group of 18:0-ACP specific SADs. However, they are divergent for the group of 16:0-ACP specific SADs. Only one amino acid residue of L115 in RcSAD1 is identical for all SADs tested here. This alignment indicates that 10 GhSADs may be 18:0-ACP specific while the other seven GhSADs possibly be 16:0-ACP specific. Further 3D modeling of GhSAD protein monomers showed that GhA-SAD6 and GhD-SAD8 had the similar structure of the substrate-bound cavity adjacent to the active diiron center with AtAAD3, but different from RcSAD1, suggesting that these two GhSADs have 16:0-ACP activity. Their substrate specificities were confirmed by our functional assay using tobacco transient expression and yeast expression system (Figure 7, 8). In contrast, GhA-SAD5 and GhA-SAD7 exhibited 18:0-ACP preference (Figure 7, 8) although they were classed into the same group of 16:0-ACP specificity by amino acid sequence alignment (Table 1). On the other hand, GhA-SAD6 and GhD-SAD8 only have 50% identity for these eight amino acid residues despite of the similar substrate specificity, just like cases of Muc-PAD, AtAAD2, and AtAAD3 with less identity but the same substrate activity. Even for RcSAD1 and Muc-PAD, only one residue was diverged for the eight amino acids between them. Taken together, these indicate that other factors may also affect the substrate specificity of SADs beyond the predominant role played by the eight amino acid residues.

In consistence with this, increasing characterization of more SADs then revealed that for substrate preference of a given chain length, some isoforms can also desaturated slightly shorter- or longer-acyl chains, despite of a reduced affinity (Kachroo et al., 2007; Rodriguez et al., 2015). Some AAD specificities were dependent on the substrate chain length. For instance, ivy (*Hedera helix*) AAD desaturated at the Δ^9^ position when presented with 18:0-ACP substrate while it catalyzed Δ^4^ desaturation when supplied with 16:0-ACP (Whittle et al., 2005). Moreover, this AAD also converted 16:1Δ^9^-ACP and 18:1Δ^9^-ACP to the corresponding Δ^4,9^ dienes. In contrast to ivy AAD, *Thunbergia* AAD produced 16:1Δ^6^ with 16:0-ACP substrate but when presented with 18:0-ACP substrate generated a mixture of Δ^6^ and Δ^9^ desaturated products (Cahoon et al., 1997). Possibly, the mechanism determining the substrate specificity of AADs may evolve independently among different plants species.

## Conclusion

The present study revealed that palmitoleic acid (16:1Δ^9^), a kind of ω-7 FAs, is specifically enriched in developing cotton endosperm, possibly benefiting the embryo development. Total of 17 GhSADs are identified in upland cotton genome and their expressions are spatiotemporally diverse, indicating that they may function differentially. Particularly, *GhA-SAD6* and *GhD-SAD8* are preferentially expressed in the developing endosperm whereas *GhA-SAD2* and *GhD-SAD6* are restricted to the embryo. Our functional characterizations using transient expression in tobacco leaves and heterologous expression in yeast mutant *BY4389* provide the first evidence showing that GhA-SAD6 and GhD-SAD8 are 16:0-ACP Δ^9^ desaturases responsible for production of 16:1Δ^9^ in the developing cotton endosperm while GhA-SAD5 and GhA-SAD7 act as 18:0-ACP Δ^9^ desaturases accounting for 18:1Δ^9^ biosynthesis in this tissue. In addition, polypeptide sequence alignment and 3D modeling of the protein monomers of these GhA-SAD6 and GhD-SAD8 suggested that their 16:0-ACP substrate specificities may be determined by the eight amino acid residues lining the bottom part of their substrate channels which distinguish them from the archetype 18:0-ACP Δ^9^ desaturase. Taken together, this study brought new insights into the SAD-mediated biosynthesis and differential accumulation of special lipid reserve in developing cotton seeds. The identified GhA-SAD6 and GhD-SAD8 could be good endogenous genes for engineering to produce higher oil enriched with ω-7 FAs in cotton seeds or other oil crops for food and industrial applications.

## Data Availability

No datasets were generated or analyzed for this study.

## Author Contributions

BL, JX, XJ, and RL designed the experiments and drafted the manuscript. BL and YS did the vector construction, fatty acid analysis and material plantation. BL and XM carried out the expression and RNA-seq analysis. RL and XJ revised the manuscript.

## Conflict of Interest Statement

The authors declare that the research was conducted in the absence of any commercial or financial relationships that could be construed as a potential conflict of interest.

## Footnotes

1 http://pfam.xfam.org/

2 https://www.cottongen.org/

3 http://smart.embl-heidelberg.de/

4 http://www.ncbi.nlm.nih.gov/Structure/cdd/cdd.shtml

5 http://mascotton.njau.edu.cn/info/1054/1118.htm

6 http://bioinformatics.psb.ugent.be/webtools/ plantcare/

7 http://arabidopsis.org/

8 http://www.ncbi.nlm.nih.gov/

9 https://swissmodel.expasy.org/
