# Supplementary material for: Stearoyl-ACP Δ9 Desaturase 6 and 8 (GhA-SAD6 and GhD-SAD8) Are Responsible for Biosynthesis of Palmitoleic Acid Specifically in Developing Endosperm of Upland Cotton Seeds
Source: Front Plant Sci. 2019 May 31;10:703. doi: 10.3389/fpls.2019.00703 (PMC6554319; doi:10.3389/fpls.2019.00703)
Supplement: Supplementary file 4 [file Table_4.docx]

| **Names** | **Cis-elements** | **Position** | **Sequences** | **Score** | **Functions** |
| --- | --- | --- | --- | --- | --- |
| GhA-SAD1 | GCN4_motif | 539 | TGAGTCA | 7 | *cis*-regulatory element involved in endosperm expression |
|  | Skn-1_motif | 285；996 | GTCAT | 5 | *cis*-acting regulatory element required for endosperm expression |
| GhA-SAD2 | Skn-1_motif | 260；1041 | GTCAT | 5 | *cis*-acting regulatory element required for endosperm expression |
| GhA-SAD4 | Skn-1_motif | 84；1143；579；159；755 | GTCAT | 5 | *cis*-acting regulatory element required for endosperm expression |
| GhA-SAD5 | Skn-1_motif | 344 | GTCAT | 5 | *cis*-acting regulatory element required for endosperm expression |
| GhA-SAD6 | Skn-1_motif | 542；1265；1094；1332，913；1221 | GTCAT | 5 | *cis*-acting regulatory element required for endosperm expression |
| GhA-SAD7 | Skn-1_motif | 51；1112；766；358，937 | GTCAT | 5 | *cis*-acting regulatory element required for endosperm expression |
| GhA-SAD9 | GCN4_motif | 915 | TGTGTCA | 7 | *cis*-regulatory element involved in endosperm expression |
|  | Skn-1_motif | 662 | GTCAT | 5 | *cis*-acting regulatory element required for endosperm expression |
| GhD-SAD1 | Skn-1_motif | 315；1091 | GTCAT | 5 | *cis*-acting regulatory element required for endosperm expression |
| GhD-SAD2 | GCN4_motif | 1480 | TGAGTCA | 7 | *cis*-regulatory element involved in endosperm expression |
|  | Skn-1_motif | 61；1218；877 | GTCAT | 5 | *cis*-acting regulatory element required for endosperm expression |
| GhD-SAD3 | Skn-1_motif | 91；226 | GTCAT | 5 | *cis*-acting regulatory element required for endosperm expression |
| GhD-SAD5 | GCN4_motif | 341 | TGTGTCA | 7 | *cis*-regulatory element involved in endosperm expression |
|  | Skn-1_motif | 344；1120 | GTCAT | 5 | *cis*-acting regulatory element required for endosperm expression |
| GhD-SAD6 | GCN4_motif | 235 | TGTGTCA | 7 | *cis*-regulatory element involved in endosperm expression |
|  | Skn-1_motif | 238 | GTCAT | 5 | *cis*-acting regulatory element required for endosperm expression |
| GhD-SAD7 | Skn-1_motif | 141 | GTCAT | 5 | *cis*-acting regulatory element required for endosperm expression |
| GhD-SAD8 | GCN4_motif | 820 | TGTGTCA | 7 | *cis*-regulatory element involved in endosperm expression |
|  | Skn-1_motif | 96；558；287；111；441 | GTCAT | 5 | *cis*-acting regulatory element required for endosperm expression |

TABLE S4 | *Cis*-elements related to endosperm expression in promoters of *GhSAD* gene family
